# Chitosan and cottonseed processing method association on carcass traits and meat quality of feedlot lambs

**DOI:** 10.1371/journal.pone.0242822

**Published:** 2020-11-23

**Authors:** Tamires da Silva Magalhães, Edson Mauro Santos, José Esler de Freitas Júnior, Stefanie Alvarenga Santos, Douglas dos Santos Pina, Luis Gabriel Alves Cirne, Luis Fernando Batista Pinto, Gerson Barreto Mourão, Franklin Delano dos Santos Soares, Laudí Cunha Leite, Henry Daniel Ruiz Alba, Manuela Silva Libanio Tosto, Gleidson Giordano Pinto de Carvalho

**Affiliations:** 1 Department of Animal Science, Federal University of Bahia, Salvador, Bahia, Brazil; 2 Department of Animal Science, Federal University of Paraiba, Areia, Paraíba, Brazil; 3 Institute of Biodiversity and Forestry, Federal University of Western Pará, Santarém, Pará, Brazil; 4 Department of Animal Science, University of São Paulo, Piracicaba, São Paulo, Brazil; 5 Department of Animal Science, University of Southwestern Bahia, Itapetinga, Bahia, Brazil; 6 Department of Animal Science, Federal University of Recôncavo da Bahia, Cruz das Almas, Bahia, Brazil; Universidade Federal de Viçosa, BRAZIL

## Abstract

The objective of this study was to evaluate the effects of the association of cottonseed processing method with chitosan on carcass traits and meat quality of lambs finished in feedlot. Eighty lambs with an average body weight of 20.6 kg, with 04 months of age, were distributed in a completely randomized design, in a 2 x 2 factorial arrangement. The factors were represented by two cottonseed processing method (whole or ground) and two levels of chitosan (0 and 136 mg/kg BW). The association of cottonseed processing method with chitosan in the lamb diet did not affect (P>0.05) carcasses traits. The pH, color, cooking losses, shear force, and proximate composition of meat were also not affected (P>0.05) by the processing method of cottonseed or its association with chitosan in the lamb diets. There was an increase in palmitoleic (*c*9-C16:1; P = 0.01) and conjugated linoleic (P = 0.02) fatty acids when ground cottonseed was associated with chitosan. Ground cottonseed associated with chitosan increases the concentration of unsaturated fatty acids in the meat of feedlot lambs.

## Introduction

People from various regions of the world are changing their dietary habits in view of concerns related to health aspects and food origins [1]. Red meat is an essential nutrient source for human health, and its physical, chemical, and biological properties determine its nutritional quality. Carcass traits and meat quality are major factors to improve the marketing of lamb meat, as these are the attributes that will determine the value of the end product [2].

Cottonseed is a relevant alternative feedstuff used in feedlots, as it combines high levels of protein (>18%) and energy (around 90% of total digestible nutrients) [3, 4]. Whole cottonseed can improve the nutritional value of lamb meat due to its high content of unsaturated fatty acids (over 70%), which are composed mainly of linoleic (C18:2n-6; 53.2%) and oleic (t9-C18:1; 17.1%) acids, as well as low saturated fatty acid content (below 3%) [5]. In addition, cottonseed can be offered ground or whole, which may affect the availability of nutrients [6] and consequently modify the final carcass traits and meat quality.

The grinding process promotes homogenization of the feed and reduction of feed selection by the animals. Additionally, grinding could increase the availability of nutrients in cottonseed for the ruminal environment. With respect to lipids, when rapidly released, they can be toxic to ruminal microorganisms [7–9], affecting growth and meat production.

Chitosan is widely used as an antimicrobial agent and, as a result, it is also largely employed as an additive in the diet of dairy herds. Its use is supported by the fact that it is considered a modulator of ruminal fermentation [10]. Chitosan has also been described to possibly optimize feed efficiency by reducing ruminal methane and increasing propionic acid production [11], as well as by inhibiting ruminal biohydrogenation and increasing vaccenic acid (*t*11-C18:1) and total conjugated linoleic acid (CLA) in *in vitro* assays [12]. However, studies investigating chitosan as a dietary additive for beef cattle herds are scarce.

To avoid an undesirable fatty acid profile in lamb meat with the use of whole or ground cottonseed [13, 14], it is hypothesized that this feedstuff can be associated with chitosan, due to its potential to reduce or prevent biohydrogenation. As a result, losses of unsaturated fatty acids—important nutrients for human health—from cottonseed in the rumen could be prevented. Therefore, we believe that the combination of cottonseed and chitosan improves meat quality by increasing its polyunsaturated fatty acid content.

This study examines the effect of associating whole or ground cottonseed with chitosan in the diet of feedlot lambs on quantitative and qualitative traits of their carcass and meat quality.

## Materials and methods

This experiment was approved by the Ethics Committee on Animal Research of the School of Veterinary Medicine and Animal Science at the Federal University of Bahia (approval no. 16/2016).

### Location, animals, experimental design, and management

The experiment was conducted at the Experimental Farm of the School of Veterinary Medicine and Animal Science at the Federal University of Bahia (EMEVZ-UFBA), located in São Gonçalo dos Campos—BA, Brazil (12°23’57.51” S and 38°52’44.66” W).

Before the experiment began, the lambs were dewormed, vaccinated against rabies and clostridial infections, and supplemented (ADE vitamin complex). Eighty male Santa Inês lambs (4 months old; 22.60 ± 2.20 kg of initial body weight) were housed in individual covered pens with suspended slatted floors (1 m^2^ per pen), equipped with feeders and drinkers.

The animals were randomly assigned to the treatments in a completely randomized design with a 2 × 2 factorial arrangements. Lambs were kept in the feedlot during 90 days, which were preceded by 15 days of adaptation to the facilities, diets, and daily management. During this phase, the animals were fed Tifton-85 hay and increasing levels of concentrate and chitosan according to random assignment to diets. The adaptation to the feed was as follows: 80:20 + 50 (roughage:concentrate + mg chitosan/kg of BW; days 1–5); 60:40 + 100 (days 6–10); and 50:50 + 136 (days 11–15). The cottonseed was ground 30 min prior to the preparation of the diet, using a mill (Nogueira® DPM 4, Itapira, Brazil) with a 5-mm sieve.

Diets were formulated as recommended by the NRC [15] to meet the nutritional requirements of lambs with an average daily gain (ADG) of 200 g/d. The roughage-to-concentrate ratio was 50:50. Treatments (Table 1) were as follows: 1) Diet containing whole cottonseed; 2) Diet containing whole cottonseed + 136 mg chitosan/kg BW; 3) Diet containing ground cottonseed; and 4) Diet containing ground cottonseed + 136 mg chitosan/kg of BW. Chitosan had a deacetylation degree of 86.3%, an apparent density of 0.33 mg/mL, and a pH of 7.9 (Polymar^®^, Fortaleza, Ceará, Brazil). The feed was supplied twice daily, at 09h00 and 16h00. The diets were weighed on a digital scale and were provided to allow approximately 10% orts (as-fed basis).

**Table 1:** Proportions and chemical composition of the basal diet used for feedlot-finished lambs.

****Ingredient (g/kg DM)****	****Diet****
Tifton-85 hay	500
Ground corn	184
Soybean meal	145
Cottonseed	150
Urea	6.00
Mineral premix^€^	15.0
**Chemical composition (g/kg DM)**	
Dry matter (g/kg as fed)	865
Organic matter	951
Ash	48.9
Crude protein	172
Ether extract	46.1
Neutral detergent fiber	417
Acid detergent fiber	209
Hemicellulose	209
Cellulose	178
Lignin	29.7
Total carbohydrates	729
Non-fibrous carbohydrates	312
Metabolizable energy (Mcal/kg) ^¥^	2.45
**Fatty acid composition (%)**	
C14:0	0.61
C15:0	0.31
C16:0	29.72
C16:1	0.22
C17:0	0.87
C17:1	0.09
C18:0	3.93
C18:1n-9	14.60
C18:2n-6	29.16
C18:3n-3	18.32
C22:0	0.63
C24:1	1.54

^€^Assurance levels (per kg in active elements): Ca: 120 g; P: 87 g; Na: 147 g; S: 18 g; Cu: 590 mg; Co: 40 mg; Cr: 20 mg; Fe: 1800 mg; I: 80 mg; Mg: 1300 mg; S: 15 mg; Zn: 3800 mg; Mo: 300 mg; F: 870 mg; phosphorus solubility in 2*%* citric acid, minimum– 95%.

^¥^Estimated according to NRC (2001).

The dry matter (DM, method 967.03), ash (method 942.05), crude protein (CP, method 981.10), and ether extract (EE, method 920.29) contents of all samples of feedstuffs and orts were determined following procedures described by the AOAC [16]. Neutral detergent fiber (aNDFom-NDF) was measured according to Van Soest *et al*. [17]. Acid detergent fiber (ADF) and lignin concentrations were determined according to AOAC (method 973.18) [18]. Hemicellulose was calculated as the difference between NDF and ADF; and cellulose as the difference between ADF and lignin. Total carbohydrates (TC) were estimated as proposed by Sniffen *et al*. [19]. The concentration of non-fibrous carbohydrates (NFC) was determined as described by Mertens [20] with modifications proposed by Hall [21]. The metabolizable energy (ME) of the diets was calculated according to NRC [22].

### Slaughter, carcass data and meat samples

At the end of the experiment (day 91), after a 16-h fasting period, the animals were weighed to obtain the final body weight. Immediately afterwards, the lamb were moved (07h00) to a commercial slaughterhouse at a distance of 30 km from the farm (60 min, approximately), at a stocking density of 0.40 m^2^/lamb during transportation. At the time of slaughter, the animals were weighed to obtain slaughter weight and desensitized by stunning with an electric discharge at the atlanto-occipital joint, followed by bleeding, skinning, and evisceration. All the procedures followed guidelines for management and humane slaughter of animals [23].

Carcasses were weighed to determine hot carcass weight (HCW) and hot carcass yield (HCY = hot carcass weight/body weight at slaughter*100) and then transferred to a cold chamber at 6°C, where they remained for 24 h. Subsequently, the carcasses were weighed again to determine cold carcass weight (CCW).

The pH measures were taken in triplicate, at the time of slaughter (approximately 45 min) and 24 h later at the left *longissimus dorsi* muscle, using a digital pH meter (HI 99163m HANNA) equipped with a penetration electrode. The device was calibrated with buffer solutions with pH 4.01 and 7.01.

After weighing, the carcasses were subjectively evaluated for conformation (1-poor, 2-fair, 3-good, 4-very good, and 5-excellent), fat cover (1-very lean, 2-lean, 3-medium, 4-fat and 5-very fat) and fatness (1-little, 2-medium, 3-great amount).

Morphometric measures were taken as follows: internal and external lengths of the carcass; length and circumference of the leg; width of rump and thorax; and circumference of rump and chest. The measurements of length and circumference were taken using a measuring tape, whereas the circumference and depth measurements were obtained using a manual measuring stick. Carcasses were divided lengthwise into two halves. The left half was sectioned into five commercial cuts to evaluate the regional composition of the carcass, namely, neck (between the first and the seventh cervical vertebra), shoulder (scapula, humerus, and carpus), ribs (between the first and the 13th thoracic vertebra), loin (bones and muscles that comprise the lumbar vertebrae), and leg (between the last lumbar and the first sacral vertebra). The right and left loins from each animal were packed, labeled, and frozen (–20°C) for later analyses.

Loin-eye area (LEA) was determined by a making transverse section between the 12th and 13th thoracic vertebrae and outlining the area corresponding to the cranial portion of the loin, using a transparency sheet. The following measurements were made: length (A) and maximum depth (B) of the *longissimus dorsi* muscle, in cm, which were obtained using a ruler. Loin-eye area was calculated by the following ellipse formula proposed by Silva Sobrinho [24]: LEA = (A/2*B/2) π, in cm^2^. Subcutaneous fat thickness (SFT) was measured in mm, using a digital caliper, at a ¾ distance from the medial side of the *longissimus* muscle towards the spinous process.

### Meat physicochemical analyses

The loins were thawed inside plastic bags at 10°C for 12 h and then dissected using scalpels. Subsequently, the color was determined in the right loins at the *longissimus dorsi* muscle. This evaluation was assessed using a colorimeter (Minolta CR—400) based on the CIELAB color system [L * (lightness), a * (red intensity), and b * (yellow intensity)]. The colorimeter was calibrated with a white ceramic plate and illuminant C, 10°, for standard observation, and it was operated using open cone. Before analysis, samples were exposed to room temperature for 30 min for the formation of oxymyoglobin. After this time, and as described by Miltenburg *et al*. [25], the L*, a*, and b* coordinates were measured in three distinct points of the internal muscle surface, and the average of the triplicates of each coordinate was calculated per animal sample.

Cooking loss (CL) was determined in each loin sample with approximately 1.5 cm thickness, 3.0 cm length, and 2.5 cm width. Raw samples were weighed, placed in an aluminum-coated tray, and cooked in a preheated oven at 170°C until the center of the meat reached a temperature of 70°C, which was measured using a copper-constantan thermocouple equipped with a digital reader. Samples were subsequently cooled at room temperature (27°C for five minutes, approximately) and re-weighed. Cooking losses were calculated as the weight difference before and after heat treatment [26]. Shear force (SF) was determined using the same cooked meat samples that were previously used for cooking losses using a Warner- Bratzler Shear Force device (3000, G- R Manufacturing CO) with a 25-kgf load cell and at a cross-head speed of 20 cm/min. Each sample was cut into three 25 × 25 mm cubes and sectioned in the transverse direction of the muscle fibers to determine meat tenderness. A texture analyzer was used in adopting the method described by Wheeler *et al*. [27], with results expressed in N/cm^2^.

Meat samples were lyophilized for 72 h and then ground in a ball mill to generate the laboratory sample for chemical composition analyses. The centesimal composition (moisture, protein, and fat) and collagen were determined by near-infrared spectroscopy [28] in 180 g of the *longissimus dorsi* muscle free of backfat, using the diagnostic tool FoodScan^TM^ (FOSS, Hillerod, Denmark) with Artificial Neural Network Calibration Model and Associated Database. The ash content was determined after ignition of a weighed sample in a muffle oven at 550°C (method 942.05) [14].

### Fatty acid profile

The composition of the fatty acids present in the lipid extract was obtained using 7 g muscle tissue collected from the *longissimus dorsi* muscle after dissection. The extraction of total lipids from muscle tissue and ingredients diets followed the methodology proposed by Hara and Radin [29], and the transesterification was performed according to Christie [30].

Fatty acid methyl esters (FAME, %) in the meat and diet ingredients were determined by gas chromatography using a chromatograph (Focus CG- Finnigan) with a flame ionization detector (FID) and a capillary column (CP-Sil 88, Varian; 100 m in length, 0.25 μm inner diameter, 0.20-μm-thick film). Hydrogen was used as the carrier gas at a flow rate of 1.8 ml/min. The program was as follows: initial oven temperature set to 70°C, held 4 min; 175°C (13°C/min), held 27 min; 215°C (40°C/min), held 9 min; and a final ramp at 7°C/min to 230°C, held 5 min, totaling 65 min. The vaporizer temperature was 250°C and the detector temperature was 300°C, following the temperature program described by Ribeiro *et al*. [31].

An aliquot of 1 μl esterified extract was injected into the chromatograph and the individual fatty acids were identified by comparing the retention times of the methyl esters presented by the Supelco™ Component FAME Mix chromatography standard (cat 18,919 Supelco, Bellefonte, PA). The fatty acid concentrations were determined by the percentage of the area of a determined fatty acid when added to the areas of all the peaks present in the sample. The results were expressed as g/100 g of total fatty acid methyl esters identified.

### Statistical analysis

Data were subjected to analysis of variance (ANOVA) in a completely randomized design. To test the effect of treatments, the data were analyzed by the MIXED procedure of SAS software [32], according to the following model:

$$Y_{ijk}=\mu +S_{i}+T_{j}+\left(S_{i}\times T_{j}\right)+e_{ijk},$$

where μ = mean; S_i_ = effect of cottonseed processing form i (i = whole vs. ground); T_j_ = effect of chitosan addition level j (j = 0 vs. 136); S_i_ × T_j_ = interaction effect between cottonseed processing form and chitosan addition; and e_ijk_ = residual error.

Treatment means were obtained by the LSMEANS procedure, adopting the significance level of 5% for all variables.

## Results

### Carcass traits

No differences were observed (P>0.05) between the treatments for slaughter weight, HCW, CCW, HCY, LEA, SFT, or commercial cuts yield. The subjective measurements of conformation, fat cover and fatness of the carcasses, as well as the morphometric measurements of external and internal lengths of the carcasses, leg length and girth, rump and thorax width, and rump, chest and thorax circumference too were not affected (P>0.05) by diets (Table 2), which may be explained by the similar slaughter weights and carcass weights. The addition of 136 mg of chitosan/kg of BW to the diet resulted in a lower proportion of shoulder (P = 0.04) as compared with the diet without chitosan.

**Table 2:** Quantitative traits, commercial cuts yields, and subjective and morphometric measurements of carcasses of lambs fed diets with cottonseed (whole or ground) associated or not associated with chitosan.

Item	Cottonseed		Chitosan€		SEM¥	P-value†		
	Whole	Ground	0	136		P	C	P×C
**Quantitative traits**								
Slaughter weight	39.19	40.70	40.24	39.65	0.524	0.18	0.60	0.40
Hot carcass weight, kg	17.30	17.76	17.58	17.48	0.241	0.36	0.86	0.25
Cold carcass weight, kg	17.17	17.66	17.46	17.37	0.238	0.31	0.85	0.27
Hot carcass yield, %	43.18	43.08	43.08	43.19	0.184	0.79	0.78	0.39
Loin-eye area, cm^2^	12.59	12.07	12.43	12.21	0.221	0.69	0.93	0.66
Subcutaneous fat thickness, mm	1.60	1.60	1.50	1.70	0.090	0.33	0.50	0.70
**Commercial cuts yield (%)**								
Neck	10.26	10.23	10.23	10.30	0.114	0.95	0.79	0.51
Shoulder	18.63	18.91	19.00	18.54	0.111	0.21	0.04	0.19
Rib	26.93	26.81	26.83	26.91	0.163	0.71	0.80	0.14
Loin	15.00	14.72	14.74	14.98	0.133	0.31	0.37	0.98
Leg	15.01	14.72	14.75	14.98	0.113	0.30	0.39	0.95
**Subjective measurements**								
Conformation	2.88	2.93	2.97	2.84	0.027	0.51	0.07	0.11
Fat cover	2.79	2.89	2.86	2.81	0.039	0.14	0.62	0.36
Fatness	2.25	2.08	2.21	2.14	0.066	0.30	0.97	0.86
**Morphometric measurements (cm)**								
Internal length	58.47	58.24	58.68	57.84	0.275	0.70	0.94	0.85
External length	53.72	53.89	54.24	53.34	0.257	0.97	0.75	0.64
Leg length	40.42	49.86	40.34	38.46	0.238	0.86	0.91	0.16
Leg circumference	38.53	38.74	38.62	39.63	0.271	0.23	0.15	0.58
Rump width	48.19	48.18	48.61	47.69	0.377	0.55	0.59	0.26
Thorax width	72.31	72.18	72.34	72.11	0.327	0.79	0.90	0.86
Rump circumference	15.83	16.11	16.01	15.84	0.107	0.58	0.40	0.50
Chest circumference	16.48	16.71	16.50	16.72	0.119	0.25	0.22	0.51
Thorax circumference	25.20	24.93	24.95	25.19	0.119	0.85	0.96	0.16

^€^mg/kg of body weight

^¥^SEM = Standard error of the mean

†Probability value for the effects of cottonseed processing (P), chitosan (C), and P × C interaction.

### Physicochemical and centesimal composition

The pH measured immediately (6.55) and 24 h after slaughter (5.88); color at the L* (36.78), a* (21.42) and b* (5.57) coordinates; cooking losses (CL; 15.54%); and shear force (SF; 23.6 N/cm^2^) were not altered (P>0.05) by the use of cottonseed (whole or ground) and chitosan.

Similarly, the chemical composition (moisture, 72.47%; ash, 1.35%, and protein, 3.71%) and collagen content (2.00%) of the *longissimus dorsi* muscle were not altered (P>0.05) by the diets (Table 3). However, a higher fat proportion (P = 0.01) was observed in the *longissimus dorsi* of the animals that did not receive chitosan, as compared with those that received a diet with 136 mg of chitosan/kg of BW.

**Table 3:** pH, color, cooking losses, shear force, centesimal composition and collagen content of the *longissimus dorsi* muscle of lambs fed diets with cottonseed (ground or whole) associated or not associated with chitosan.

Item	Cottonseed		Chitosan€		SEM¥	P-value†		
	Whole	Ground	0	136		P	C	P×C
pH_45min_	6.56	6.54	6.57	6.53	0.273	0.13	0.29	0.66
pH_24h_	5.85	5.92	5.95	5.82	0.038	0.38	0.09	0.49
Lightness (L*)	36.99	36.57	36.62	36.94	0.328	0.50	0.62	0.43
Red intensity (a*)	21.55	21.30	21.45	21.39	0.196	0.52	0.90	0.64
Yellow intensity (b*)	5.75	5.39	5.47	5.67	0.199	0.37	0.61	0.76
Cooking losses, %	14.37	16.70	16.09	15.00	0.631	0.22	0.57	0.23
Shear force, N/cm^2^	23.10	24.10	24.50	22.70	0.837	0.91	0.39	0.65
**Centesimal composition (%)**								
Moisture	72.27	72.68	72.57	72.38	0.198	0.30	0.62	0.17
Ash	1.29	1.41	1.25	1.45	0.064	0.28	0.05	0.05
Protein	22.44	21.90	22.08	22.26	0.109	0.29	0.67	0.46
Fat	3.73	3.49	4.08	3.55	0.099	0.39	0.01	0.06
Collagen	2.01	1.99	2.01	1.99	0.049	0.91	0.93	0.83

^€^mg/kg of body weight

^¥^SEM = Standard error of the mean

†Probability value for the effects of cottonseed processing (P), chitosan (C) and P × C interaction.

### Fatty acid profile

The concentration of palmitic acid (C16:0) was higher (P = 0.01) in the meat of the lambs fed ground cottonseed (Table 4).

**Table 4:** Fatty acid composition (percentage of total fatty acids) of the *longissimus dorsi* muscle of lambs fed diets with cottonseed (ground or whole) with or without the addition of chitosan.

Item	Cottonseed		Chitosan€		SEM¥	P-value†		
	Whole	Ground	0	136		P	C	P×C
****ƩSaturated fatty acids (ƩSFA)****	****49.62****	****50.39****	****50.12****	****49.89****	****0.358****	****0.70****	****0.99****	****0.93****
C6:0	0.01	0.00	0.01	0.01	0.001	0.91	0.91	0.88
C8:0	0.09	0.01	0.01	0.01	0.001	0.27	0.53	0.60
C10:0	0.17	0.17	0.17	0.17	0.003	0.28	0.73	0.24
C12:0	0.14	0.14	0.13	0.14	0.007	0.97	0.50	0.01
C14:0	2.54	2.95	2.85	2.90	0.071	0.53	0.89	0.27
C15:0	0.38	0.38	0.38	0.37	0.009	0.67	0.43	0.07
C16:0	22.84	23.27	22.88	22.79	0.157	0.01	0.77	0.91
C17:0	0.82	0.81	0.81	0.78	0.115	0.52	0.47	0.79
C18:0	22.50	22.54	22.77	22.60	0.355	0.95	0.68	0.67
C20:0	0.05	0.03	0.04	0.04	0.002	0.25	0.69	0.77
C22:0	0.08	0.08	0.07	0.08	0.004	0.87	0.40	0.95
****ƩMonounsaturated fatty acids (ƩMUFA)****	****44.25****	****43.31****	****43.87****	****43.65****	****0.280****	****0.06****	****0.48****	****0.22****
*c*9-C14:1	0.08	0.06	0.07	0.07	0.002	0.68	0.95	0.21
*c*9-C16:1	1.42	1.40	1.43	1.39	0.021	0.56	0.30	0.01
C17:1	0.22	0.21	0.22	0.22	0.003	0.94	0.50	0.40
*c*9-C18:1	36.43	35.33	35.89	35.84	0.282	0.43	0.79	0.57
*c*11-C18:1	2.11	2.07	2.16	2.02	0.073	0.80	0.32	0.20
*c*12-C18:1	1.09	1.07	1.10	1.07	0.033	0.71	0.69	0.27
*c*13-C18:1	0.65	0.63	0.66	0.62	0.019	0.51	0.31	0.15
*c*15-C18:1	0.14	0.14	0.14	0.14	0.005	0.68	0.87	0.45
*t*9-C18:1	1.56	1.85	1.69	1.74	0.068	<0.01	0.58	0.12
*t*16-C18:1	0.46	0.46	0.46	0.47	0.008	0.46	0.87	0.16
C20:1	0.03	0.04	0.04	0.03	0.002	0.15	0.64	0.50
****ƩPolyunsaturated fatty acids (ƩPUFA)****	****6.13****	****6.30****	****6.01****	****6.46****	****0.189****	****0.37****	****0.08****	****0.99****
C18:2n-6	3.97	4.06	3.90	4.16	0.108	0.51	0.53	0.58
****Ʃn-3****	****0.48****	****0.52****	****0.49****	****0.51****	****0.017****	****0.26****	****0.53****	****0.77****
C18:3n-3	0.22	0.25	0.24	0.24	0.001	0.06	0.16	0.59
C20:5n-3	0.10	0.10	0.10	0.11	0.006	0.92	0.57	0.66
C22:5n-3	0.21	0.20	0.19	0.22	0.011	0.65	0.79	0.70
C22:6n-3	0.03	0.03	0.03	0.03	0.008	0.53	0.78	0.98
****Ʃn-6****	****4.91****	****5.00****	****4.65****	****5.26****	****0.160****	****0.76****	****0.06****	****0.57****
C18:3n-6	0.00	0.01	0.00	0.00	0.001	0.47	0.71	0.96
C20:2n-6	0.01	0.02	0.01	0.01	0.001	0.11	0.57	0.64
C20:4n-6	1.03	1.01	0.96	1.08	0.049	0.75	0.55	0.69
****n-6/n-3****	****9.22****	****9.09****	****9.03****	****9.30****	****0.004****	****0.32****	****0.10****	****0.33****
****PUFA/SFA****	****0.13****	****0.13****	****0.12****	****0.13****	****0.017****	****0.27****	****0.52****	****0.77****
****Conjugated linoleic acid (CLA)**‡**	****0.60****	****0.56****	****0.56****	****0.61****	****0.015****	****0.16****	****0.18****	****0.02****

^€^mg/kg of body weight

^¥^SEM = Standard error of the mean

†Probability value for the effects of cottonseed processing (P), chitosan (C) and P × C interaction.

‡CLA = *c*9*t*11-C18:2 + *t*7*c*9-C18:2

There was an interaction effect (P = 0.01) between the ground cottonseed and chitosan on the proportion of lauric acid (C12:0), resulting in a lower concentration of this fatty acid in the meat of lambs fed diets with ground cottonseed and without chitosan.

The *t*9-C18:1 (elaidic) fatty acid was affected by cottonseed processing (P<0.01), since the animals fed ground cottonseed showed a higher proportion of this fatty acid in the *longissimus dorsi*.

There was an interaction effect (P = 0.01) between cottonseed and chitosan (Table 5) on the proportion of palmitoleic acid (*c*9-C16:1). While the association of ground cottonseed with chitosan increased the proportion of this fatty acid, the opposite effect was observed when only whole cottonseed was offered, inducing a decrease in the levels of this fatty acid.

**Table 5:** Interactions between cottonseed processing and chitosan on fatty acids (percentage of total fatty acids) in the *longissimus dorsi* muscle of lambs.

Decomposition of interactions		
Cottonseed	Chitosan	
	0	136
	**C12:0 fatty acid**	
Whole	0.15^aA^	0.13^aA^
Ground	0.11^bA^	0.16^aA^
SEM	0.013	0.015
	***c*9-16:1 fatty acid**	
Whole	1.50^aA^	1.35^bA^
Ground	1.37^bA^	1.43^aA^
SEM	0.038	0.046
	**Conjugated linoleic acid (*c*9*t*11-C18:2 + *t*7*c*9-C18:2)**	
Whole	0.58^aA^	0.55^aA^
Ground	0.55^bA^	0.66^aA^
SEM	0.028	0.033

Means followed by different letters (lowercase in the row and uppercase in the column) differ statistically (P<0.05) according to the F test.

There was no effect of diets on the concentrations of omega-3 (n-3) and omega-6 (n-6) or PUFA/SFA ratio in the *longissimus dorsi* muscle of the lambs. However, there was an interaction effect (P = 0.02) between the cottonseed and chitosan on the proportion of the conjugated linoleic acid (CLA; *c*9*t*11-C18:2 + *t*7*c*9-C18:2), with the association between ground cottonseed and chitosan increasing its concentration in the meat (Table 5).

## Discussion

### Carcass traits

Similar results were found by Paim *et al*. [14], who tested the inclusion of 19.5% cottonseed (whole cottonseed or high-oil cottonseed meal) in lamb diets. The authors observed that cottonseed did not affect quantitative traits of Santa Ines lambs, which showed an average HCY of 44.2%.

Chitosan is known for its effect on ruminal fermentation, where it modifies the volatile fatty acid production patterns, improving the efficiency of energy utilization for growth and production [33]. However, in this study, slaughter weight was similar between the animals, suggesting that the chitosan dose supplied did not influence animal performance. Similar results were obtained for HCW and CCW.

Shoulder yield was lower (P = 0.04) in the group fed the diet with chitosan. The results found in this study for the yields of neck, shoulder, rib, and loin are similar to those observed by Pereira *et al*. [34] in feedlot lambs fed a diet containing 15% cottonseed.

The yields of the commercial cuts of the carcass observed in this study are in agreement with the theory of anatomical harmony described by Boccard and Drumond [35], which states that carcasses with similar fat contents (Table 3) exhibit almost all body regions in similar proportions, regardless of the genotype.

### Physicochemical and centesimal composition

The meat pH values were close to the 6.70 and 5.60 found by Campos *et al*. [36] and the 6.45 and 5.70 reported by Cirne *et al*. [37], at 0 and 24 h after slaughter, respectively. Thus, the pH results of the present study indicate that there possibly was a typical development of *rigor mortis* and the inexistence of pre-slaughter stress [38–40], suggesting the absence of alterations in meat quality [41].

The centesimal composition values of the *longissimus dorsi* muscle in this study (moisture, 72.47%; ash, 1.35%; protein, 22.17% and fat, 3.71%) was close to those reported in the literature for feedlot lambs [36, 42, 43].

In the present experiment, the addition of chitosan promoted a decrease in the meat fat content (P = 0.01). This effect can be explained by the characteristic of chitosan to selectively bind to specific organic compounds such as cholesterol, proteins, fats, and triglycerides, making them unavailable [44] and thus influencing their absorption, which results in decreased body fat.

### Fatty acid profile

The higher passage rate of ground cottonseed [7] associated with the increased EE digestibility provided by the inclusion of chitosan in the diet [9] possibly increased the lauric acid (C12:0) content in the meat. Cottonseed diets have a high content of SFA [14], which have antiprotozoal and antibacterial effects during ruminal fermentation [45–47], promoting changes in the end products, as in the fatty acid profile [48].

Saturated fatty acids are associated with human health problems [49–51]. However, lauric acid (C12:0) prevents cardiovascular disease by reducing the oxidation of LDL, further increases the HDL levels in the blood, reduces blood pressure and triggers apoptosis in cancer cells [52].

Although the palmitic acid (C16:0) found in foods is often associated with adverse effects on chronic diseases in adults, this fatty acid can be elongated to stearic acid (C18:0), considered neutral. This fatty acid, in turn, is converted to oleic acid (*c*9-C18:1) through the Δ9-desaturase enzyme. In addition, it is an essential component of the membrane, secretory, and transporting lipids, having crucial roles in protein palmitoylation and signaling molecules [53–55].

It is possible that chitosan could modulate characteristics of ruminal fermentation, improving the fatty acid profile of meat [12]. The chitosan effect is related to increased desaturation of palmitoleic acid and palmitic acid [56]. These events were higher in the meat of the lambs fed ground cottonseed (Table 4), and this was also possibly due to the larger contact surface between cottonseed and chitosan, preventing the action of ruminal microorganisms. When cottonseed was included in the lamb diets [13], there was a reduction of palmitoleic acid in the meat of the animals (1.37% in control diet vs. 0.17% in the diet with 30% cottonseed inclusion).

Studies that examined the mechanism of action of palmitoleic acid (*c*9-C16:1) have described that it regulates lipogenesis and coordinates the systemic metabolism [57, 58]. Palmitoleic acid acts as the insulin-sensitizing hormone that improves glucose metabolism, reducing weight gain, adipose tissue deposition, and circulating levels of insulin. Additionally, it reduces *de novo* lipogenesis and increases the oxidation of fatty acids, increasing energy expenditure and its storage [59–61]. These results reveal the importance of this fatty acid for the human diet, as it could improve the quality of life and life expectancy.

The larger contact surface of ground cottonseed for the ruminal microorganisms and its association with chitosan likely contributed to the increase in CLA. This corroborates another study [12] in which researchers evaluated the *in vitro* effect of chitosan on the biohydrogenation of unsaturated fatty acids.

The decrease in biohydrogenation and increased amount of unsaturated fatty acids in the rumen and meat as induced by chitosan are uncertain [12]. However, these changes would improve the nutritional properties of ruminant-derived food products, especially by increasing CLA. This would reflect positively on human health and well-being, given the benefits provided by this fatty acid [62–64].

## Conclusion

The use of cottonseed, whole or ground, in association with chitosan in diets for feedlot lambs does not change their slaughter weight, carcass yield, or meat quality. However, dietary inclusion of ground cottonseed associated with chitosan (136 mg/kg BW) improves the fatty acid profile and nutritional quality of lamb meat, as it induces a 20.4% increase in the concentration of conjugated linoleic acid (CLA), a fatty acid beneficial to human health.
